# Postarrest Care Bundle Improves Quality of Care and Clinical Outcomes in the Normothermia Era

**DOI:** 10.1177/08850666231223482

**Published:** 2024-01-04

**Authors:** Andrew J. Caddell, Dave Nagpal, Ahmed F. Hegazy

**Affiliations:** 1 Cardiology Division, 3688 Dalhousie University, Halifax, Nova Scotia, Canada; 2 Critical Care, 6221 Western University, London, Ontario, Canada

**Keywords:** cardiac arrest, temperature management, quality improvement, normothermia

## Abstract

**Purpose:**

Temperature targets in patients with cardiac arrest and return of spontaneous circulation (ROSC) have changed. Changes to higher temperature targets have been associated with higher breakthrough fevers and mortality. A post-ROSC normothermia bundle was developed to improve compliance with temperature targets.

**Methods:**

In August 2021, “ad hoc” normothermia at the discretion of the attending intensivist was initiated. In December 2021, a post-ROSC normothermia protocol was implemented, incorporating a rigorous, stepwise approach to fever prevention (temperature ≥ 37.8). We conducted a before-after cohort study of all adult patients post-ROSC who survived to intensive care unit admission between August 1, 2021, and April 1, 2022. They were divided into “ad hoc” and “protocol” groups. Clinical outcomes compared included fevers, active cooling, and paralytic use.

**Results:**

Fifty-eight post-ROSC patients were admitted; 24 in the “ad hoc” and 34 in the “protocol” groups. Patient demographics were similar between groups. The “ad hoc” group had more shockable rhythms (67% vs 24%, *P* = .001) and cardiac catheterizations (42% vs 15%, *P* = .03). The “protocol” group were significantly less likely to have a fever at 40 h (6% vs 40%, *P* < .001) and 72 h (14% vs 65%, *P* ≤ .001). Patients in the normothermia “protocol” used significantly less neuromuscular blocking agents (24% vs 50%, *P* = .05). The normothermia “protocol” resulted in similar mortality (56% vs 58%, *P* = 1.0).

**Conclusion:**

Use of a normothermia “protocol” resulted in fewer fevers and less neuromuscular blocker administration compared to “ad hoc” management. A protocolized approach for improved quality of care should be considered in institutions adopting normothermia.

## Introduction

All comatose cardiac arrest survivors are likely to benefit from some form of temperature control.^1,2^ The optimal temperature post-ROSC (return of spontaneous circulation) is unknown. Earlier randomized controlled trials (RCTs), such as the HACA^3^ and Bernard^4^ trials, suggested benefit to therapeutic hypothermia (targeting 32-34 °C) in patients with shockable out-of-hospital cardiac arrest (OHCA). Subsequently, the TTM trial demonstrated no significant difference between mild and moderate hypothermia (33 °C vs 36 °C).^5^ This prompted a recommendation by the Canadian Cardiovascular Society in 2017 stating: “We recommend that a temperature between 33 °C and 36 °C, inclusively, be selected and maintained for patients who undergo TTM (Strong Recommendation; Moderate-Quality Evidence).”^2^

TTM2 was published in 2021; the largest RCT to date examining temperature management post cardiac arrest.^6^ Therein, patients were randomized to normothermia (target temperature less than 37.5 °C with active cooling if temperature exceeded 37.7) versus hypothermia (33 °C) in patients with OHCA. This rigorous study failed to show a significant difference between a hypothermic and normothermic approach, with similar mortality and neurologic outcomes. Subsequent meta-analysis^7^ and network meta-analysis^8^ supported an absence of benefit to therapeutic hypothermia.

Outside of clinical trials, observational studies auditing higher temperature targets have been associated with poorer compliance, increased fevers and a trend toward reduced survival.^9^ In an attempt to improve compliance with new temperature targets, we developed a new post-ROSC normothermia protocol and a nursing guideline document. Implementation of post cardiac arrest care bundles has previously been demonstrated to improve survival and functional recovery.^10^ Our primary goal was to reduce fevers at 40 h (similar to the duration of the TTM2 trial). Our secondary endpoint was fever reduction at 72 h.

## Methods

London Health Sciences Centre (LHSC) is an academic healthcare institute providing tertiary and quaternary care to adults in Southwestern Ontario. Within its catchment area, all adults with cardiac arrest and ROSC are admitted to intensive care units (ICUs) at 2 campuses of LHSC. The University Hospital contains the Medical Surgical ICU (MSICU, 38 beds, approximately 1500 admissions/ year), and the Victoria Hospital contains the Critical Care Trauma Centre (CCTC, 35 beds, approximately 1500 admissions/ year). Both campuses admit cardiac arrest patients have cardiology consultative services and cardiac catheterization labs. Although patient composition is similar, solid-organ transplant patients are siloed at the MSICU and obstetrics, trauma, and hematology are siloed at CCTC. London Health Sciences Centre uses an electronic medical record (EMR) and electronic medical order entry system.

### Patient Population

All patients with cardiac arrest with ROSC who are unresponsive are admitted to the one of the 2 LHSC campuses (CCTC or MSICU). All admissions are tracked in the EM. Two of the study coordinators were automatically notified of all ROSC patient admissions. Patients were excluded if they were <18 years old, temperature control was not pursed by the treatment team or they did not survive to ICU admission. All adult ROSC patients were eligible for inclusion.

### Ad Hoc Normothermia

In August 2021, consultant-driven “ad hoc” normothermia commenced. Temperature targets, temperature duration, sedation, and cooling mechanisms were at the complete discretion of the attending intensivist.

### Normothermia Protocol

In November to December 2021, we developed a post-ROSC normothermia protocol emphasizing a stepwise approach to fever prevention for 72 h (temperature target <37.5 °C). The working group included intensive care physicians, neurologists with special interest in neurocritical care, cardiologists, respiratory therapists, and nursing managers. Two plan-do-study-act (PDSA) cycles were planned. Prior to the PDSA, emails were sent soliciting feedback by the intensive care attending staff.

Protocol modifications occurred in 2 rapid PDSA cycles:

PDSA 1 (November 23-29, 2021)—A trial of the online order set with intensive care nurses, respiratory therapists, and physicians was done to identify potential issues with order set initiation. This was live tested on a simulated electronic patient using the electronic order system. Following corrections to incorrect orders and nursing documents, it was approved for use by the ICU joint counsel (nurse educators, ICU managers, and physicians).
PDSA 2 (December 16-21, 2021)—Index trial of order set with 3 patients was done to identify remaining errors and discrepancies related to order set initiation. Feedback was solicited from practitioners using them in real time. In addition, discrepancies in the nursing protocols were corrected.

The final iteration of the post-ROSC normothermia protocol was released for institution-wide implementation on December 21, 2021. Ahead of implementation, instruction emails were sent and information sessions were held with intensive care physicians and staff. A full timeline is presented in Table 1. Patients with temperatures below 37 °C received no cooling therapy. Patients with temperatures at or above 37 °C received cold saline, ambient cooling devices, ice packs, and paralytics. All patients in the normothermia “protocol” received high-dose sedation for 40 h. Sedation between hours 40 and 72 was at the discretion of the attending intensivist. The full protocol is attached as Appendix A.

**Table 1.** Project Timeline.

Time	Event
August 2021	Beginning of “Ad Hoc” Normothermia at LHSC
	Beginning of study period
November 2021	REB approval for study
	Normothermia Protocol developed
	First PDSA cycle
December 2021	Second PDSA cycle
	Normothermia Protocol released
April 2022	End of study period

Abbreviations: LHSC, London Health Sciences Centre; PDSA, plan-do-study-act.

### Data Source and Collected Characteristics

We conducted a before-after cohort study of all post-ROSC patients in the “ad hoc” and “protocol” groups between August 1, 2021, and April 1, 2022. Demographic information was extracted from the EMR, including age, sex, comorbidities, arrest details, and cardiac interventions, performed. Temperature targets, duration, and cooling modalities were extracted for the “ad hoc” group. Our primary outcome was fever within the study period (at 40 h and 72 h). The EMR was also used to extract information about fevers (defined as temperature ≥37.8 °C), medications used, morbidity (Modified Rankin Score, MRS, assessed at discharge), hospital duration, and mortality. Shock was defined as a need for vasopressors and a lactate >4 mmol, regardless of etiology. All patients had esophageal probes for hourly temperature monitoring in the post-ROSC period.

### Statistical Analysis

Continuous variables were expressed as means with standard deviations for normally distributed data and medians with interquartile ranges for non-normally distributed continuous variables. Categorical variables were expressed as percentages. Fisher exact test was used to compare categorical variables. Means were compared using *t* tests. Medians were compared using the Mann-Whitney *U* test. The Western University Research Ethics Board approved this study. The Strobe Checklist was completed with this manuscript.

## Results

Over the 8-month period, 58 post-ROSC patients were admitted; 24 in the “ad hoc” and 34 in the “protocol” groups. Complete data were present for all patients until discharge. Patient demographics are documented in Table 2. Patients in the “ad hoc” group had more shockable rhythms (67% vs 24%, *P* = .001) and more cardiac catheterizations (42% vs 15%, *P* = .03).

**Table 2.** Post-ROSC Patient Demographics in Ad Hoc and Protocol Normothermia.

	Ad hoc N = 24	Protocol N = 34	*P* Value
Age (year), mean (SD)	60 (12)	63 (16)	.44
Male sex, n/total N (%)	15/24 (63%)	25/34 (74%)	.40
MSICU campus, n/total N (%)	15/24 (63%)	18/34 (53%)	.59
Hypertension, n/total N (%)	16/24 (66%)	17/34 (50%)	.28
Dyslipidemia, n/total N (%)	11/24 (45%)	12/34 (35%)	.59
Diabetes, n/total N (%)	11/24 (45%)	11/34 (32%)	.41
Prior MI, n/total N (%)	9/24 (38%)	6/34 (18%)	.13
Prior CABG, n/total N (%)	2/24 (8%)	1/34 (3%)	.56
Prior CHF, n/total N (%)	6/24 (25%)	7/34 (21%)	.76
IHCA, n/total N (%)	8/24 (33%)	11/34 (33%)	1.00
Shockable rhythm, n/total N (%)	16/24, (67%)	8/34 (24%)	.001
Duration of arrest, min (SD)	24 (18)	17 (10)	.063
Cardiac etiology, n/total N (%)	15/24 (63%)	13/34 (38%)	.11
Respiratory etiology, n/total N (%)	8/24 (33%)	14/34 (41%)	.59
STEMI, n/total N (%)	5/24 (20%)	2/34 (6%)	.11
Cardiac catheterization, n/total N (%)	10/24 (42%)	5/34 (15%)	.03
pH post-ROSC, mean (SD)	7.12 (0.17)	7.09 (0.19)	.54
Lactate post-ROSC, mean (SD)	7.7 (4.9)	8.7 (4.4)	.42
Shock post-ROSC, n/total N (%)	19/24 (79%)	27/34 (79%)	1.00

Abbreviations: MSICU, medical surgical intensive care unit; ROSC, return of spontaneous circulation.

All patients in the “protocol” group had clearly defined temperature targets and temperature durations established in the order set. Significantly fewer patients in the “ad hoc” group had clear orders defining temperature targets (66% vs 100%, *P* < .001) and target temperature duration (54% vs 100%, *P* < .001). “Ad hoc” chosen temperature ranges varied significantly (34-36 °C, 35-36 °C, 36-37 °C, 36-37.5 °C, <37.5 °C, and <38 °C).

Patient outcomes are demonstrated in Table 3. Patients in the “protocol” group were significantly less likely to have a fever at 40 h (6% vs 40%, *P* ≤ .001) and 72 h (14% vs 65%, *P* < .001) compared to those treated with “ad hoc” normothermia. In addition, significantly less patients in the normothermia “protocol” received paralytics to maintain target temperature (24% vs 50%, *P* = .05). The normothermia “protocol” resulted in similar mortality (56% vs 58%, *P* = 1.0). The normothermia “protocol” resulted in similar survival with good neurological outcome, defined as MRS < 3 (21% vs 29%, *P* = .45).

**Table 3.** Post-ROSC Patient Outcomes in the “Ad Hoc” and the “Protocol” Groups.

	Ad Hoc N = 24	Protocol N = 34	*P* Value
Fever within 40 h, n/total N (%)	10/14 (42%)	2/34 (6%)	<.001
Fever within 72 h, n/total N (%)	16/24 (66%)	5/34 (14%)	<.001
% measurements febrile within 40 h, mean (SD)	4% (7.6)	0.29% (1.2)	.012
% measurements febrile within 72 h, mean (SD)	4% (5.4)	0.61% (1.8)	.006
Hospital LOS, median (IQR)	8 (17)	7 (6)	.30
Mortality, n/total N (%)	14/24 (58%)	19/34 (56%)	1.0
Modified Rankin Score, median (IQR)	6 (5)	6 (2)	.92
Modified Rankin scale 0-2, n/total N (%)	7/24 (29%)	7/34 (21%)	.45
Paralytic use, n/total N (%)	12/24, (50%)	8/34 (24%)	.05
Active cooling, n/total N (%)	18/24 (75%)	26/34 (76%)	1.0

Abbreviations: IQR, interquartile range; ROSC, return of spontaneous circulation.

## Discussion

In this single-centered study in an academic center, “ad hoc” normothermia was associated with high rates of fevers and paralytic use. The normothermia protocol was developed expeditiously, refined through 2 PDSA cycles, and then rolled out for implementation at 2 ICU campuses. This normothermia protocol remains the post-arrest protocol at LHSC.

Protocols and bundles have robust evidence in critically ill patients,^11^ operating theaters,^12^ and postcardiac arrest patient management.^13–15^ This protocolization of care minimizes heterogenicity between providers. In our study, patients with “ad hoc” temperature management frequently did not have temperature orders or targets, potentially leading to confusion within the healthcare team. When providers defined targets in the “ad hoc” group, chosen temperature ranges varied significantly (34-36 °C, 35-36 °C, 36-37 °C, 36-37.5 °C, <37.5 °C, and <38 °C), as did duration (24 h, 40 h, 48 h, and 72 h). By contrast, all patients in the “protocol” group had temperature targets and durations clearly defined in the orders. Patients in the “protocol” group spent significantly more time in the target temperature range. Patients in the “protocol” group had less cardiac etiologies for their cardiac arrests. We are unaware of any literature suggesting etiology of arrest influencing frequency or severity of fever. We felt that early cardiac catheterization was unlikely to negatively influence temperature control, as the earliest fever in the post-PCI group occurred 7 h following the procedure. Anecdotally, support staff and trainees reported less confusion with uniform temperature treatment post cardiac arrest.

TTM2^6^ and subsequent meta-analyses^7,8^ have suggested normothermia post-ROSC and therapeutic hypothermia at minimum have clinical equipoise. As authors, we believe this is not synonymous with therapeutic nihilism in regard to temperature control. Although patient outcomes were similar between groups, it is noteworthy that the protocolized normothermia group contained significantly more “non-cardiac” etiologies for arrest and this is a group who would be expected to have worse outcomes. Furthermore, despite the better temperature control in the “normothermia” group, there was significantly less paralytic use. Use of paralytics in other conditions such as acute respiratory distress syndrome and sepsis has been associated with ICU acquired weakness, including critical illness polyneuropathy, or critical illness myopathy.^16^ This may have been because of earlier temperature interventions and universal deep sedation in the “protocol” group. An optimal sedation strategy post-ROSC has yet to be elucidated. Strengths of this protocol include its rapid adaptation, flexibility, clear instructions to nursing staff, and broad inclusion.

This study has several limitations. First, before-after study designs lend themselves to the possibility of unmeasured confounding and secular trends. In addition, during the period of “protocol” preparation, there may have been inadvertent crossover in the run-in period. Secondly, this was a single-center trial. Findings would therefore be more generalizable to centers with similar practice standards. Thirdly, the sample sizes in both groups were small and were underpowered for significant clinical outcomes (such as mortality). The “ad hoc” data were retrospective. The marked differences in group compositions are likely reflective of these small sample sizes.

## Conclusion

Use of a normothermia protocol in post-ROSC patients resulted in fewer fevers and less paralytic use compared to “ad hoc” temperature management. Despite a greater proportion of “ad hoc” patients having shockable rhythms and cardiac catheterization, mortality was similar. Institutions desiring change to post-ROSC normothermia should consider adopting a protocolized approach for improved quality of care.

## Supplemental Material

sj-docx-1-jic-10.1177_08850666231223482 - Supplemental material for Postarrest Care Bundle Improves Quality of Care and Clinical Outcomes in the Normothermia Era Supplemental material, sj-docx-1-jic-10.1177_08850666231223482 for Postarrest Care Bundle Improves Quality of Care and Clinical Outcomes in the Normothermia Era by Andrew J. Caddell, MD, FRCPC, Dave Nagpal, MD, FRCSC, and Ahmed F. Hegazy, MD, FRCPC in Journal of Intensive Care Medicine
