# Supplementary material for: Postarrest Care Bundle Improves Quality of Care and Clinical Outcomes in the Normothermia Era
Source: J Intensive Care Med. 2024 Jan 4;39(7):623–7. doi: 10.1177/08850666231223482 (PMC11149385; doi:10.1177/08850666231223482)
Supplement: sj-docx-1-jic-10.1177_08850666231223482 - Supplemental material for Postarrest Care Bundle Improves Quality of Care and Clinical Outcomes in the Normothermia Era [file sj-docx-1-jic-10.1177_08850666231223482.docx]

Appendix A - Protocol for Normothermic (35C to 37C Degrees) TTM

**Protocol for Normothermic (35C to 37C Degrees) Targeted Temperature Management (TTM) in Critical Care Post Cardiac Arrest**

This protocol provides instructions to health care providers for the initiation, maintenance and discontinuation of NORMOTHERMIC (35 to 37C Target) Temperature Management (TTM) in the Adult Critical Care Units, London Health Sciences Centre (LHSC).

For patients being treated with hypothermic TTM, use the Protocol for Hypothermic TTM.

The goal is to reach the target temperature as quickly as possible after ROSC. Fever avoidance (temperature less than 37.1C) is extremely important. About half of patients who are being treated with normothermic TTM may still require active cooling to keep their temperature within target. This protocol should only be used in patients who are not spontaneously following commands.

Maintain target temperature if investigations are required. Patients should not be allowed to waken or breathe spontaneously; if they do, sedation and analgesia is inadequate. This checklist covers the 72 hours of TTM protocol.

Central venous and arterial access should be established for rapid and secure vascular access for the potential administration of cold fluids, vasoactive agents and/or pacemaker insertion. Cooling *should not be delayed* for the insertion of a CVC/arterial line.

| **Time Points (in Hours):**  **T1:** Time point when patient first achieves a temperature below 37.1C  **T40:** Time point for the end of the period of deep sedation  **T72:** End of targeted temperature management period  **T1 – T40:** Time period for the maintenance of deep sedation and temperature less than 37.1C  **T40-T72:** Fever prevention period; keep temperature less than 37.1C. If temperature exceeds target, sedation and/or other cooling methods may need to be reinstituted. | |
| --- | --- |
| **INITIATION OF NORMOTHERMIC (35C TO 37C) TTM (T1 to T40): PROTOCOL REQUIREMENTS** |  |
| - If patient has indications of an acute STEMI, Code STEMI should be activated. Temperature control is not a contraindication to coronary intervention. |  |
| - If patient has cardiogenic shock or persistent arrythmia, the Critical Care Consultant/Senior Resident should engage Cardiology STAT for cardiac catheterization consideration. |  |
| - Administer ASA, antiplatelet agents, anticoagulant and/or fibrinolytics as per orders for Acute Coronary Syndrome. |  |
| - Target Temperature: 35C to 37C |  |
| - Standard analgesia orders should be discontinued and replaced with BOTH analgesic and sedative orders contained within the TTM protocol to provide anaesthetic level dosing. |  |
| **STEPS TO ACHIEVING NORMOTHERMIA** |  |
| - Notify RRT STAT to ensure full controlled ventilation is established. Switch to non-heated humidification if temperature greater than 36C. |  |
| - Quickly perform a BRIEF baseline neurological assessment including GCS, pupils, corneal reflex, gag and cough, then PROMPTLY initiate sedation by bolus administration. |  |
| - Initiate continuous esophageal temperature monitoring via nasally inserted probe [(probe insertion instructions](about:blank)) and document hourly. A bladder or pulmonary artery catheter can also be used for core temperature monitoring. Rectal temperature monitoring can be used temporarily but can be less accurate (particularly if stool is present). Monitor temperature continuously and document hourly until T72. |  |
| - Administer a liberal bolus dose of narcotic and sedative. If temperature is greater than 37C, **administer an initial 250 mL cold saline bolus immediately** **following narcotic and sedative administration.** Do not delay cooling until infusions are initiated. |  |
| - Repeat narcotic bolus and initiate infusions (start cooling as soon as first bolus dose is given; do not wait until infusion is started). Titrate narcotic and sedative infusions to ensure the following:   - No response to a tap on the forehead   - MAAS 0   - CPOT 0   - No respiratory effort   **Patients should NOT be allowed to waken or trigger the ventilator during first 40 hours.** |  |
| - **Repeat 250 mL** cold normal saline bolus using 250 - 500 mL refrigerated bags (to ensure solution remains cold to end of infusion) to a maximum of 1000 mL if temperature remains greater than 37C. |  |
| - Once above criteria is maintained with narcotic and sedative infusions, administer a bolus dose of neuromuscular blocking agent if necessary for temperature management or ventilator control. Not all normothermia patients will require saline or neuromuscular blockage. Rocuronium is preferred (cost) unless severe renal or hepatic impairment. |  |
| - Repeat neuromuscular blocking agent bolus and/or initiate an infusion if MAAS 0 and CPOT 0 *AND* patient is making respiratory effort/triggering ventilator, shivering or if target temperature remains above goal. Always treat with narcotic or sedative first to ensure anesthetic levels of sedation. |  |
| - If temperature is greater 37C, place cooling blanket over TOP surface of patient. Place a light sheet between the patient and blanket (no cooling blanket under patient (pressure injury risk) or directly in contact with skin (frost bite risk). |  |
| - Wrap arms and legs in a flannelette blanket to prevent shivering and frost-bite if cooling blanket is required. |  |
| - Add ice packs around neck, in axilla and groin if temperature remains greater than 37C. |  |
| - If patient is receiving CRRT, turn heater to off. |  |
| - If temperature remains above target OR there is any other suspicion for sepsis, patient should be pan cultured. Review need for empiric antimicrobials with provider. |  |
| - Identify the time point when target temperature is first achieved. This will be T1. |  |
| - If patient's temperature is spontaneously below target, do not initiate active warming unless the temperature is less than 32C. Discontinue warming devices when temperature reaches 33C. |  |
| - If triggering on the ventilator is noted or patient shows any signs of responsiveness or movement, bolus the patient with additional narcotic and increase the maintenance infusions of sedatives until deep anaesthesia is achieved. Do not allow the patient to lighten during first 40 hours. |  |

| **SEIZURE MONITORING**   - Initiate continuous EEG (CEEG) (for all patients on normothermia protocol). Continue until T72, patient is awake or discontinued by provider (whichever comes first). - Consult Neurocritical care (Monday to Friday) if seizure activity is suspected or myoclonus observed. Consult Neurology on evenings and weekends. - Neurocritical care is able to view CEEG information from the central station from either campus. If you identify something concerning on the CEEG, identify the exact time of the event to help Neurocritical care isolate the CEEG finding in full disclosure. - Although not 100% reliable, an SEF of less than 4 suggests that the patient is deeply sedated. - The preferred treatment for myoclonus is valproate. CCTC physicians may order the first dose but Neurology or Neurocritical care consultation is required for ongoing approval. - Note that neuromuscular blocking agents are not anticonvulsants but they can mask detection of a motor seizure. |
| --- |
| **MONITORING, MAINTENANCE AND NURSING CARE** |
| - Send TSH for patients who are spontaneously hypothermic at admission. |
| - If it is difficult to maintain target temperature of 35C to 37C or there are any other signs/risk factors for possible sepsis, culture patient at admission and review need for empiric antimicrobial therapy with provider. |
| - If a patient’s temperature is spontaneously below 35C, do not actively re-warm unless temperature is less than 32C. Stop active rewarming when temperature reaches 33C. |
| - Review MAP target with provider (65 for most patients). To date, there has not been an optimum MAP target in the post-arrest patient. |
| - Ensure that ECG, electrical cords and/or pacemaker cables do not come in contact with wet linen |
| - Continue with routine turning and skin care. Monitor for myocardial irritability during position changes. |
| - Monitor closely for signs of pressure injury. Do not place cooling blanket under patient (increases pressure injury risk). |
| - For neurological vital signs, monitor pupils and CEEG changes only until T40, then resume full neuro assessment (pupils should respond when neuromuscular blocking agents are in use). |
| - Monitor for signs of frostbite/dusky circulation. |
| - Keep eye lids closed at all times and provide lacrilube **ointment** per order. |
| - If anti-arrhythmics are required, amiodarone is the usual first line agent unless contraindicated. |
| - Initiate enteral feeding upon admission. Small bowel placement is preferred. Initiate gastric feeding at 10 ml/hr (elevated HOB as tolerated). |
| - Initiate DVT and GI prophylaxis as per standard care. |

| **END OF SEDATION AND FEVER PREVENTION PERIOD (T40 to T72)** |
| --- |
| - Continue to measure core temperature continuously and document hourly until T72 |
| - If neuromuscular blockers are in use, stop infusion at T40 if temperature is < 37C. |
| - Stop narcotic and sedative infusions 2 hours after stopping neuromuscular blocking agents if temperature is greater than 35C and less than 37.1C. Review timing with provider if patient is in renal failure and received rocuronium. |
| - Review sedation/analgesia administration goals with provider if temperature remains less than or equal to 35C at T48. |
| - Once infusions are discontinued, continue with PRN narcotics and sedatives as required for symptom management or temperature control. |
| - If temperature exceeds 37C between T40 and T72 and patient is NOT fully awake, reintroduce cooling in the following sequence to maintain temperature below 37C:   1. Minimize clothing/linen   2. Administer regular dose acetaminophen as ordered   3. Use non-heated humidification on the ventilator circuit and CRRT circuit.   4. Restart sedation until temperature is within target or VAMASS 0 (lowest dose required)   5. Initiate cooling blanket   6. Add ice packs in axilla and groin   7. Initiate neuromuscular blockade |

| **ACTIVE REWARMING (IF REQUIRED) FOR T48 TO T72** |
| --- |
| - If temperature remains less than 35C at T48 active warming with an external surface warming device may be initiated |
| - Turn warmer off as soon as temperature reaches 36C |

**POST PROTOCOL**

- Continue CEEG until order received to discontinue
- Most patients with persistent coma at 72 hours will require neuroimaging (CT head, MRI brain) and formal EEG
- Patients should have assessment of cardiac function (formal echocardiogram or POCUS)
- Review with team the need for Neuro Critical Care Consult (usually at 72 hours or later)
- Neurologic prognostication should be delayed until 96 hours or later

Appendix B

STROBE Statement—checklist of items that should be included in reports of observational studies

|  | Item No | Recommendation |
| --- | --- | --- |
| **Title and abstract** | 1 | (*a*) Indicate the study’s design with a commonly used term in the title or the abstract. *Done.* |
|  |  | (*b*) Provide in the abstract an informative and balanced summary of what was done and what was found. *Done.* |
| Introduction | | |
| Background/rationale | 2 | Explain the scientific background and rationale for the investigation being reported. *Done.* |
| Objectives | 3 | State specific objectives, including any prespecified hypotheses *Done.* |
| Methods | | |
| Study design | 4 | Present key elements of study design early in the paper. *Done.* |
| Setting | 5 | Describe the setting, locations, and relevant dates, including periods of recruitment, exposure, follow-up, and data collection. *Done.* |
| Participants | 6 | (*a*) *Cohort study*—Give the eligibility criteria, and the sources and methods of selection of participants. Describe methods of follow-up. *Done.*  *Case-control study*—Give the eligibility criteria, and the sources and methods of case ascertainment and control selection. Give the rationale for the choice of cases and controls  *Cross-sectional study*—Give the eligibility criteria, and the sources and methods of selection of participants |
|  |  | (*b*) *Cohort study*—For matched studies, give matching criteria and number of exposed and unexposed.  *Case-control study*—For matched studies, give matching criteria and the number of controls per case |
| Variables | 7 | Clearly define all outcomes, exposures, predictors, potential confounders, and effect modifiers. Give diagnostic criteria, if applicable. *Done.* |
| Data sources/ measurement | 8* | For each variable of interest, give sources of data and details of methods of assessment (measurement). Describe comparability of assessment methods if there is more than one group. *Done.* |
| Bias | 9 | Describe any efforts to address potential sources of bias. *Done.* |
| Study size | 10 | Explain how the study size was arrived at. *Done.* |
| Quantitative variables | 11 | Explain how quantitative variables were handled in the analyses. If applicable, describe which groupings were chosen and why. *Done.* |
| Statistical methods | 12 | (*a*) Describe all statistical methods, including those used to control for confounding. *Done* |
|  |  | (*b*) Describe any methods used to examine subgroups and interactions. *N/A* |
|  |  | (*c*) Explain how missing data were addressed. *N/A.* |
|  |  | (*d*) *Cohort study*—If applicable, explain how loss to follow-up was addressed. N/A  *Case-control study*—If applicable, explain how matching of cases and controls was addressed  *Cross-sectional study*—If applicable, describe analytical methods taking account of sampling strategy |
|  |  | (*e*) Describe any sensitivity analyses N/A. |

Continued on next page

| Results | | |
| --- | --- | --- |
| Participants | 13* | (a) Report numbers of individuals at each stage of study—eg numbers potentially eligible, examined for eligibility, confirmed eligible, included in the study, completing follow-up, and analysed. *Done.* |
|  |  | (b) Give reasons for non-participation at each stage. N/A |
|  |  | (c) Consider use of a flow diagram. N/A |
| Descriptive data | 14* | (a) Give characteristics of study participants (eg demographic, clinical, social) and information on exposures and potential confounders. *Done* |
|  |  | (b) Indicate number of participants with missing data for each variable of interest N/A |
|  |  | (c) *Cohort study*—Summarise follow-up time (eg, average and total amount). *Done* |
| Outcome data | 15* | *Cohort study*—Report numbers of outcome events or summary measures over time. *Done* |
|  |  | *Case-control study—*Report numbers in each exposure category, or summary measures of exposure |
|  |  | *Cross-sectional study—*Report numbers of outcome events or summary measures |
| Main results | 16 | (*a*) Give unadjusted estimates and, if applicable, confounder-adjusted estimates and their precision (eg, 95% confidence interval). Make clear which confounders were adjusted for and why they were included. *Done* |
|  |  | (*b*) Report category boundaries when continuous variables were categorized. *Done* |
|  |  | (*c*) If relevant, consider translating estimates of relative risk into absolute risk for a meaningful time period. |
| Other analyses | 17 | Report other analyses done—eg analyses of subgroups and interactions, and sensitivity analyses N/A |
| Discussion | | |
| Key results | 18 | Summarise key results with reference to study objectives. *Done* |
| Limitations | 19 | Discuss limitations of the study, taking into account sources of potential bias or imprecision. Discuss both direction and magnitude of any potential bias *Done* |
| Interpretation | 20 | Give a cautious overall interpretation of results considering objectives, limitations, multiplicity of analyses, results from similar studies, and other relevant evidence *Done* |
| Generalisability | 21 | Discuss the generalisability (external validity) of the study results *Done* |
| Other information | | |
| Funding | 22 | Give the source of funding and the role of the funders for the present study and, if applicable, for the original study on which the present article is based *Done* |

*Give information separately for cases and controls in case-control studies and, if applicable, for exposed and unexposed groups in cohort and cross-sectional studies.

**Note:** An Explanation and Elaboration article discusses each checklist item and gives methodological background and published examples of transparent reporting. The STROBE checklist is best used in conjunction with this article (freely available on the Web sites of PLoS Medicine at http://www.plosmedicine.org/, Annals of Internal Medicine at http://www.annals.org/, and Epidemiology at http://www.epidem.com/). Information on the STROBE Initiative is available at www.strobe-statement.org.
